# Three cases in which drug‐induced hyponatremia was improved by replacing carbamazepine with lacosamide

**DOI:** 10.1002/ccr3.2857

**Published:** 2020-04-14

**Authors:** Masahito Morimoto, Ichiro Suzaki, Seishi Shimakawa, Toshiaki Hashimoto, Tadanori Nakatsu, Shigeaki Hamada, Shojiro Kyotani

**Affiliations:** ^1^ Japanese Red Cross Tokushima Hinomine Rehabilitation Center for People with Disabilities Tokushima Japan; ^2^ Graduate School of Pharmaceutical Sciences Tokushima Bunri University Japan

**Keywords:** antiepileptic drug, carbamazepine, hyponatremia, lacosamide, side effects

## Abstract

Carbamazepine often causes drug‐induced hyponatremia. Hyponatremia due to carbamazepine may be improved by changing to the same mechanism of action, lacosamide.

## Graphical abstract

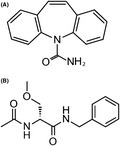

## 1 INTRODUCTION

Carbamazepine often causes drug‐induced hyponatremia. Hyponatremia presents a variety of symptoms and sometimes affects life. Hyponatremia due to carbamazepine may be improved by replacing to the same mechanism of action, lacosamide.

Among the various adverse reactions of antiepileptic drugs (AEDs) known, hyponatremia has been described to occur at a relatively high incidence with carbamazepine (CBZ).^1^ CBZ exerts its antiepileptic effect by facilitating fast inactivation of voltage‐gated sodium channels.^2^ It is an AED that requires to be used with caution because of potential drug interactions owing to its enzyme‐inducing activity and the need for appropriate dose setting based on plasma drug concentration monitoring. However, CBZ is still generally recognized as a first‐line therapeutic agent for localization‐related epilepsy on account of the abundant evidence for its excellent efficacy. ^3^

Meanwhile, lacosamide (LCM), a novel AED which inactivates the sodium channels just like CBZ, is devoid of enzyme‐inducing activity and thus requires no dosage adjustment by plasma drug concentration monitoring. LCM, therefore, is recommended as a first‐line drug for localization‐related epilepsy by US Expert Opinion 2016.^4^

We encountered 3 cases of symptomatic localization‐related epilepsy under long‐term CBZ therapy who showed amelioration of hyponatremia following treatment switch from CBZ to LCM. Herein, we present a report of these cases.

This report is being presented with the approval of the Ethics Review Committee of the Japanese Red Cross Tokushima Hinomine Rehabilitation Center for People with Disabilities. Guardians of the patients were given assurance about protection of the patients' personal information and provided informed consent in writing.

## 2 CASE REPORT

Table 1 presents the patient background characteristics and data pertaining to the frequency of seizure, and blood and urine tests before and after the AED regimen switch.

**Table 1:** Patient characteristics and change values of each item before and after replacement from CBZ to LCM

Patients		A	B	C
Age		35	28	39
Sex		Male	Female	Male
Primary disease		Cerebral palsy	Acute encephalopathy	CFC syndrome
Epilepsy classification^a^		Symptomatic localization related	Symptomatic localization related	Symptomatic localization related
Combined AEDs		LEV, PRP	LEV	LEV, PB
Base dose of CBZ (mg/day)		300	400	800
Blood concentration of CBZ (μg/mL)		5.1‐7.5	7.7‐11.2	5.2‐6.7
LCM dose after change (mg/day)		400	200	400
Average number of seizure	before （/times/M）	14.7 ± 1.52	1.67 ± 1.53	3.67 ± 1.15
	after （/times/M）	14.3 ± 2.04	1.67 ± 0.57	3.45 ± 0.86
	*P*	.42	.88	.53
Changes in weight	before (kg)	40.9‐42.3	32.4‐33.5	24.8‐25.3
	after (kg)	41.3‐42.5	31.8‐32.6	25.0‐25.6
Changes in fluid intake	before (/mL/d)	1650‐2100	1430‐1950	1350‐1630
	after (/mL/d)	1600‐2050	1530‐1900	1280‐1700
Changes in urine volume	before (/mL/d)	1470‐1920	1160‐1870	1020‐1550
	after (/mL/d)	1350‐1900	1200‐1780	1150‐1580
Blood test values				
Changes in BUN	before (mg/dL)	10.6 ± 1.24	7.90 ± 1.75	11.3 ± 0.47
	after (mg/dL)	10.2 ± 1.93	8.43 ± 0.41	11.1 ± 0.73
Changes in serum creatinine	before (mg/dL)	0.16 ± 0.01	0.22 ± 0.02	0.28 ± 0.02
	after (mg/dL)	0.15 ± 0.03	0.26 ± 0.01	0.28 ± 0.01
Changes in sodium	before (meq/L)	132.0 ± 2.82	131.5 ± 0.70	133.0 ± 1.41
	after (meq/L)	137.0 ± 2.83	138.5 ± 0.70	136.0 ± 1.41
	*P*	<.05	<.05	<.05
Changes in potassium	before (meq/L)	5.20 ± 0.21	4.26 ± 0.52	3.90 ± 0.41
	after (meq/L)	4.73 ± 0.16	4.20 ± 0.35	4.26 ± 0.23
Changes in phosphorus	before (mg/dL)	3.93 ± 0.04	3.53 ± 0.28	3.86 ± 0.12
	after (mg/dL)	3.63 ± 0.44	3.50 ± 0.14	3.73 ± 0.04
Urine test values				
Changes in protein	before	(‐) ‐ (±)	(‐)	(‐)‐(1+)
	after	(‐)‐(±)	(‐)‐(±)	(‐)‐(±)
Changes in urobilinogen	before	(±)	(±)	(±)
	after	(±)	(±)	(±)
Changes in ketone	before	(‐)	(‐)	(‐)
	after	(‐)	(‐)	(‐)
Changes in pH	before	7.66 ± 0.62	6.66 ± 0.84	7.16 ± 1.24
	after	7.90 ± 0.53	6.50 ± 1.08	6.16 ± 1.08
Changes in specific gravity	before	1.018 ± 0.004	1.006 ± 0.002	1.016 ± 0.002
	after	1.016 ± 0.002	1.013 ± 0.002	1.022 ± 0.003

Note

Statistical analysis used paired *t* test.

Abbreviations: AED, antiepileptic drug; BUN, blood urea nitrogen; CBZ, carbamazepine; CFC, cardio facio cutaneous; LCM, lacosamide; LEV, levetiracetam; M, month; *P*, *P* value; PB, phenobarbital; PRP, perampanel.

^a^ Classification of epilepsy, epilepsy syndrome, and related paroxysmal disease (ILAE1989).

The subjects were 3 hospitalized patients with long‐standing epilepsy, with an approximately 30 years' duration of the illness. All 3 patients had very severe intellectual disability and were unable to converse with or enable establishment of mutual understanding with the healthcare professionals. The underlying diseases each had central nervous system disorders with cerebral palsy, acute encephalopathy, and CFC syndrome, but internal organs such as the heart, liver, and kidney were healthy.

In all the 3 cases, the clinical classification category was symptomatic localization‐related epilepsy and the patients had been receiving AEDs since their nursing infant stage. All three received concomitant administration of levetiracetam (LEV) and had been suffering from hyponatremia from even prior to the start of concomitant LEV administration. No means for correction of the dietary sodium intake, such as administration of sodium chloride, was undertaken in any of the 3 cases. The patient had a dietitian properly managing salt and water intake. The combined drugs other than the AEDs were patient A: levocarnitine, carbocisteine, ambroxol, and epinastine; patient B: distigmine, picosulfate, and lubiprostone; and patient C: picosulfate. Their plasma sodium levels had been maintained within the range of 130‐135 mEq/L over time; hence, the degree of hyponatremia was mild. The causes of hyponatremia varied, but there were no symptoms or diseases such as vomiting, diarrhea, malignancy, lung disease, heart failure, liver failure, peripheral edema, ascites, and hyperglycemia. There was no use of diuretics, antidepressants, desmopressin, and immunoglobulins. Nephrogenic syndrome of inappropriate antidiuresis also has the same characteristics as syndrome of inappropriate secretion of antidiuretic hormone, but was considered negative in this case because it is a rare genetic disease and is more likely to develop in infancy.^5^, ^6^

Based on these findings, we suspected the use of antiepileptic drugs common to all patients, especially drug‐induced hyponatremia due to CBZ. They were followed up because patients with mild hyponatremia and mental retardation are prone to hyponatremia.^7^ However, they are patients with refractory epilepsy and switched from CBZ to LCM with the aim of improving serum sodium levels. The switch of the AED drug was carried out slowly over a period of about 3 months, so as to gradually decrease the CBZ dosage and gradually increase the LCM dosage.

The measurement of the sodium level was performed 3 times before LCM addition and after LCM reached the maintenance dose. Blood was taken at around 6 AM, with no drugs or foods. The number of seizure was observed by healthcare professionals and described in medical records daily. The average number of seizure was investigated 6 months before addition of LCM and 6 months after LCM reached the maintenance dose, and was examined from medical record and compared monthly average number.

The plasma CBZ level at the baseline during CBZ therapy was 5.1‐7.5 μg/mL in patient A, 7.7‐11.2 μg/mL in patient B and 5.2‐6.7 μg/mL in patient C. The daily dose of CBZ before the switch and that of LCM after the switch were CBZ = 300 mg and LCM = 400 mg in patient A, CBZ = 400 mg and LCM = 200 mg in patient B, and CBZ = 800 mg and LCM = 400 mg in patient C. There was no significant correlation between the dose level of CBZ and that of LCM.

No significant change was noted in any of the 3 cases with respect to the mean number of seizure during the 6‐month period before and after the drug change (patient A was *P* = .42, patient B was *P* = .88, and patient C was *P* = .53). Six months after change, plasma sodium levels improved significantly in all 3 cases (*P* < .05 for all three patients). There was no significant change in body weight, fluid intake, urine volume, blood test values (blood urea nitrogen, creatinine, potassium, and phosphorus), and urine test values (protein, urobilinogen, ketone, pH, and specific gravity) before and after the drug replacement.

## 3 DISCUSSION

In those cases, the cause of hyponatremia is likely to be CBZ. So we decided to exclude CBZ. It would be nice if there was no need to add LCM (other drugs). However, the subjects were refractory epilepsy patients who could not control seizures even with multiple AEDs, so they had to add LCM, the same sodium channel blocker as CBZ. LCM was demonstrated in a global phase III trial to be equivalent to CBZ in its epileptic seizure‐suppressing efficacy.^8^ The cases presented here do not change the number of seizures and consider that the effectiveness of LCM was comparable to that of CBZ.

With regard to the mechanism of development of hyponatremia during therapy with CBZ, it has been reported that CBZ stimulates antidiuretic hormone (ADH) secretion and that the excess ADH binds to ADH receptors of the renal collecting tubules, resulting in cyclic adenosine monophosphate (AMP) production and excessive enhancement of the water permeability of the collecting tubules.^7^

**Figure 1:**
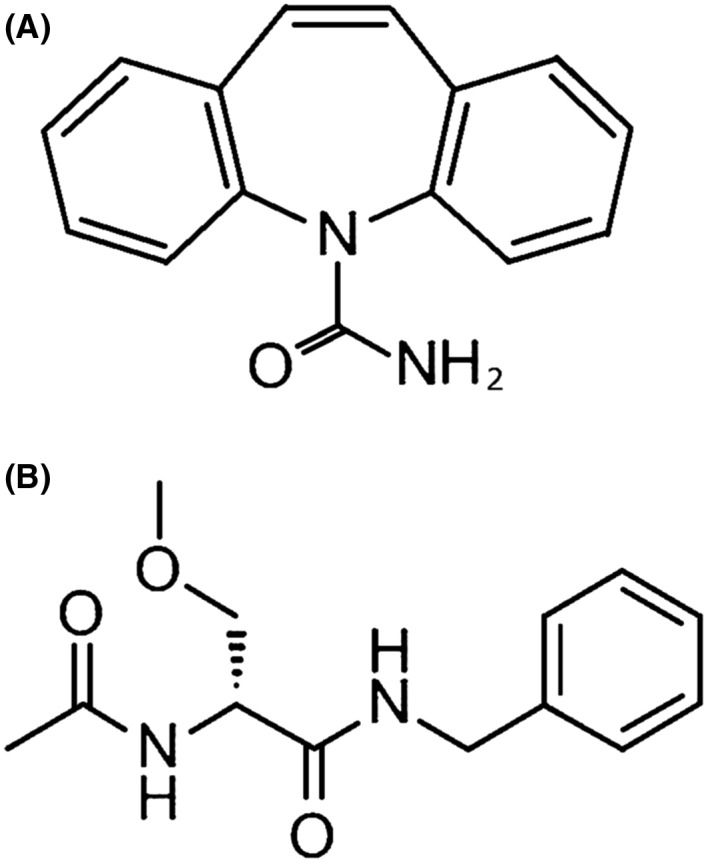
Chemical structural formula of carbamazepine and lacosamide. A, carbamazepine; B, lacosamide

Although the precise molecular mechanism by which CBZ exerts this effect remains unclear, we speculate two possibilities. First, the dibenzazepine skeleton of CBZ (Figure 1A) could account for the action. Hyponatremia has also been reported to occur frequently with oxcarbazepine, which is another AED that possesses a dibenzazepine skeleton‐like CBZ.^1^ It would seem reasonable to assume, therefore, that this skeleton may bind to some molecule involved in the production and secretion of ADH, thereby promoting ADH formation. LCM differs structurally from CBZ and consists primarily of a D‐serine (Figure 1B); it is presumed to be associated with a reduced risk of hyponatremia.

The second possibility pertains to the physicochemical properties of CBZ. CBZ, being a highly hydrophobic drug (log *P* = 1.76), is almost entirely metabolized in the liver and is known to induce hepatic metabolizing enzymes, such as the cytochrome P450 (CYP) series.^9^ Furthermore, there have been reports recently indicating that CBZ increases the plasma lipid levels and decreases plasma thyroid hormone concentrations, and the possible involvement of enzyme induction by CBZ has been pointed out.^10^, ^11^ There is the possibility that the same or a comparable mechanism may be involved in the abnormal enhancement of ADH secretion as well. LCM is a renally excreted drug with lower hydrophobicity (log *P* = .25) as compared to CBZ and has been shown not to induce hepatic metabolizing enzymes; this could be the reason why it is associated with a lower risk of hyponatremia.

The incidence of hyponatremia due to CBZ has been reported to be 1.8%‐40%.^12^ It is also known that CBZ is used in combination with clobazam, phenytoin, and valproic acid as a hyponatremia‐inducing factor.^13^ Perampanel also reports hyponatremia,^14^ but it is <1% and occurs less frequently. Levetiracetam has also been reported to induce hyponatremia.^15^, ^16^ However, in all three cases, the same amount of LEV was used before and after replacing CBZ with LCM. Therefore, this report considers that LEV was not a factor influencing serum sodium levels. Therefore, it was considered that there were no other factors besides CBZ that could contribute to hyponatremia.

As regards measures to manage hyponatremia, hyponatremia at plasma sodium levels of 130‐135 mEq/L is classified as mild according to the “Clinical Practice Guideline on Diagnosis and Treatment of Hyponatremia 2014”.^17^ It is recommended that “drugs which may cause hyponatremia be discontinued” in subjects with chronic hyponatremia presenting with no serious symptoms. It may be that the patients in the cases reported herein were incapable of complaining of subjective symptoms due to the extremely severe intellectual disability. Since the findings of long‐term follow‐up of hyponatremia cases have suggested a relationship of the condition with a poor survival prognosis,^18^ it is of cardinal importance to control the plasma sodium level.

This case series has limitations. First, ADH measurements were not performed because some data were collected from retrospective observations. Second, the hyponatremia in this case was mild. Therefore, it is necessary to confirm in the future whether the change from CBZ to LCM has a significant effect on serum sodium concentration in patients with severe hyponatremia.

## 4 CONCLUSION

Replacing from CBZ to LCM may not improve all hyponatremia. However, we have experienced that replacing to LCM is one of the ways to improve hyponatremia caused by CBZ. Therefore, for localized epilepsy, it is recommended to use a safer LCM as a first‐line drug or to switch from CBZ to LCM if the patient has hyponatremia caused by CBZ.

## CONFLICT OF INTEREST

None decalred.

## AUTHOR CONTRIBUTIONS

Suzaki: contributed to the drug treatment and examination of the target patients. Morimoto, Hashimoto, and Kyotani: extracted and analyzed patient data and considered the factors. Shimakawa, Nakatsu, and Hamada: critically reviewed and corrected the contents of medical and pharmaceutical matters.
